# Application potential of toll-like receptors in cancer immunotherapy: Systematic review

**DOI:** 10.1097/MD.0000000000003951

**Published:** 2016-06-24

**Authors:** Ming Shi, Xi Chen, Kangruo Ye, Yuanfei Yao, Yu Li

**Affiliations:** School of Life Science and Technology, Harbin Institute of Technology, Harbin, China.

**Keywords:** cancer therapy, innate immunity, therapeutic target, toll-like receptor

## Abstract

Toll-like receptors (TLRs), as the most important pattern recognition receptors in innate immunity, play a pivotal role in inducing immune response through recognition of microbial invaders or specific agonists. Recent studies have suggested that TLRs could serve as important regulators in the development of a variety of cancer. However, increasing evidences have shown that TLRs may display quite opposite outcomes in cancer development. Although several potential therapeutic Toll-like receptor ligands have been found, the mechanism and therapy prospect of TLRs in cancer development has to be further elucidated to accelerate the clinical application. By performing a systematic review of the present findings on TLRs in cancer immunology, we attempted to evaluate the therapeutic potential of TLRs in cancer therapy and elucidate the potential mechanism of cancer progress regulated by TLR signaling and the reported targets on TLRs for clinical application. An electronic databases search was conducted in PubMed, Chinese Scientific Journal Database, and Chinese Biomedical Literature Database from their inception to February 1, 2016. The following keywords were used to search the databases: Toll-like receptors, cancer therapy, therapeutic target, innate immunity. Of 244 studies that were identified, 97 nonrelevant studies were excluded. In total, 147 full-text articles were assessed, and from these, 54 were excluded as they did not provide complete key information. Thus, 93 studies were considered eligible and included in the analysis. According to the data from the included trials, 14 TLR ligands (77.8%) from 82 studies have been demonstrated to display antitumor property in various cancers, whereas 4 ligands (22.2%) from 11 studies promote tumors. Among them, only 3 TLR ligands have been approved for cancer therapy, and 9 ligands were in clinical trials. In addition, the potential mechanism of recently reported targets on TLRs for clinical application was also evaluated in this review. We show that targeting TLRs in cancer immunotherapy is a promising strategy for cancer therapy, and the specific TLR ligands, either alone or combination, exhibit antitumor potential.

## 1 Introduction

Toll-like receptors (TLRs), mammalian homolog of drosophila Toll protein, are regarded as critical pattern recognition receptors (PRRs) of innate immunity, which recognize pathogens through sensing pathogen-associated molecular patterns (PAMPs) derived from bacteria, virus, fungi, and protozoa.^[1,2]^ Each TLR contains transmembrane domain, extracellular PAMPs binding domain with leucine-rich repeats motif, and intracellular Toll-IL-1 receptor (TIR) domain that initiates signaling cascade.^[3]^ Recognition of microbial invaders by TLRs leads to activation of downstream signaling cascade to secret cytokines and chemokines and finally results in activation of both innate and adaptive immune response to clean pathogens.^[4,5]^ Additionally, TLRs also play roles in maintaining tissue homeostasis, in which TLRs regulate wound healing, including noninfections inflammation, tissue repair, and regeneration, through recognizing endogenous danger signals (Danger-associated molecular pattern, DAMPs).^[6–8]^

Emerging evidences have indicated that TLRs play important roles in cancer progress (Table 1); however, the function and biological mechanism of TLRs in cancer seems complex. Different activated TLRs may display completely opposite effects in cancer, antitumor or procarcinogenesis.^[9–12]^ The opposite outcome of TLRs activation perhaps due to the distinct TLRs and downstream signaling pathways that are activated in immune cells and/or cancer cells; or the chronological order of TLR activation in cancer cells or immune cells, which markedly affects the subsequent activation and induced effectors.^[13]^ Some TLRs on cancer cells may favor cancer progress in an inflammation-dependent or -independent way. Inflammatory response stimulated by TLR signaling could promote oncogenesis by boosting tumor inflammatory microenvironment. In addition, elevated expression levels of certain types of cancer cell TLRs also could promote tumorigenesis which is required for TLR adapter molecules, but independent of inflammation.^[14–16]^ However, some TLR agonists have been found to induce strong antitumor activity by indirectly activating tolerant host immune system to destroy cancer cells. Therefore, utilizing the specific agonists or antagonists of TLRs might represent a promising new strategy against cancer.

**Table 1:**
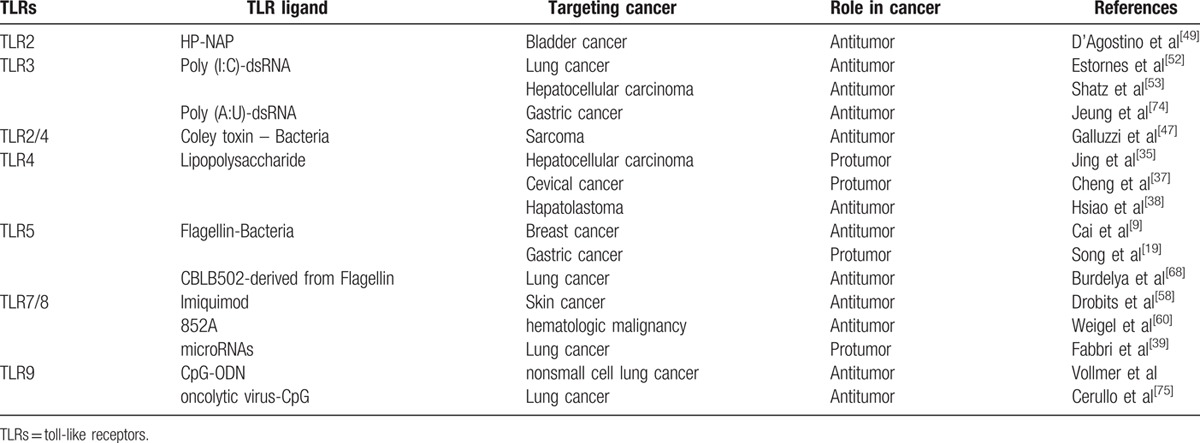
TLR ligands and roles in cancer progress.

## 2 Methods

### 2.1 Databases search

A systematic search was conducted in the following 3 online electronic databases from their inception until February 1, 2016: PubMed, Chinese Scientific Journal Database, and Chinese Biomedical Literature Database. The following keywords were used to search the databases: (“Toll-like receptor” OR “TLR”) AND (“cancer therapy” OR “cancer treatment” OR “Immunotherapy” OR “therapeutic target”) AND (“immunity” OR “innate immunity” OR “innate immune response”).

### 2.2 Search strategy and selection criterion

Two reviewers independently identified the potential literatures and selected studies in accordance with the search strategy. Then, the titles and abstracts of the articles were further screened for the potential relevance. At last, the full texts of eligible references were retrieved for further identification. Disagreements were resolved by consultation or consensus with the third reviewer.

### 2.3 Ethics approval

Ethical approval or patient consent was not needed because this is a systematic review in which all data were extracted from published literature.

## 3 Results

### 3.1 The expression and activation of TLRs in cancer cells

Increasing evidences have shown that TLRs are expressed not only in immune cells, such as macrophages and dendritic cells, but also in various tumor cells.^[17,18]^ Some TLRs in tumor cells indeed biologically impact on tumor cell growth and survival. For example, TLR5 is highly expressed in breast carcinoma and gastric carcinoma cells, and TLR5 signaling inhibits breast cancer growth, but promotes the proliferation of gastric cancer cells.^[9,12,19]^ However, it is not all the TLRs expressed in cancer cells are functional to induce inflammatory response. In breast cancer cell MDA-MB-231 and MDA-MB-435, TLR5 is abnormally localized only in cytoplasm, so NF-κB signaling cannot be activated by TLR5 agonist in these cells.^[9]^ Similarly, full-length TLR9, expressing on the cell surface, is functional to promote proliferation of HCC, whereas the cleaved forms of TLR9 are localized in cytoplasm and nonresponsive to cell surface stimulation of TLR9 agonist.^[20]^ These evidences suggest that there are different expression pattern of TLRs in tumor cells and TLRs in tumor cells are functional to activate downstream signal only if they localize at reasonable sites in cell, so that TLRs can recognize specific ligands and trigger TLR signaling.

Most ligands of TLRs are various PAMPs. TLRs that are expressed on cell surface, including TLR1, TLR2, TLR4, TLR5, and TLR6, mostly recognize bacteria and fungi PAMPs. Other TLRs (TLR3, TLR7, TLR8, TLR9) are localized within cell and sense viral RNA and CpG DNA.^[21]^ In addition to PAMP ligands, an increasing number of DAMPs are reported to associate with TLRs in cancer cells.^[22]^ Kim et al found that the endogenous versican, an extracellular matrix proteoglycan, induced inflammatory tumor microenvironment and stimulated metastasis of Lewis lung carcinoma through activation of TLR2–TLR6 complex.^[23]^ Endogenous heat-shock proteins (HSPs) are accumulated in tumor cells, and a fraction of HSPs can be released into the extracellular milieu from tumor cells. The extracellular HSPs are recognized by TLR2 and TLR4 as DAMPs, and induce acute inflammation to mediate immune suppression.^[24,25]^ Structural studies showed that TLRs did more than merely recognized specific ligands. TLRs tend to form homodimer or heterdimer, recruit other extracellular and intracellular proteins to the complex, and initiate downstream signaling cascades.^[26,27]^

TLR signaling, stimulated by specific ligands, may induce different responses in distinctive tumor cell types. Most TLR family members, except TLR3, tend to predominantly activate NF-κB via MyD88, which regulates the induction of proinflammatory cytokines. MyD88 variants may lead to reduced NF-κB-mediated inflammatory response.^[28]^ Recent studies demonstrated that activated TLRs linked with programmed cell death in cancer cells. The selective expression of low-frequently used MyD88 rendered cells much more sensitive to TLR-mediated programmed cell death instead of NF-κB pathway.^[28–30]^ Accordingly, defective NF-κB signaling led to enhanced programmed cell death induced by TLR ligands.^[30]^

### 3.2 TLRs influence inflaming metastasis of cancer

Malignant tumors are initially characterized by 6 hallmarks, including self-sufficiency in growth signals, insensitivity to antigrowth signals, evading apoptosis, limitless replicative potential, sustained angiogenesis, and Tissue invasion and metastasis.^[31]^ However, these essential capabilities do not include immune mechanism. It has become clear that inflammation links tumor microenvironment to metastasis. Thus, Alberto Mantovani proposes that inflaming metastasis is the seventh hallmark of cancer.^[32]^ All these hallmarks that acquired from genetic changes are constant and essential features of cancers. Evidences have shown that chronic inflammation induced by TLR ligands is associated with carcinogenesis; moreover, cancer cells express TLRs to facilitate inflammation, which further support tumor development and metastasis.^[33]^ Huang et al believed that TLR2 silencing might be a potential siRNA-based gene therapy for hepatocarcinoma. They found TLR2 knockdown with shRNA inhibited proliferation of hepatocarcinoma cell line BLE-7402, and decreased the secretion of cytokine IL-6 and IL-8. Mice of BLE-7402 xenograft tumor model treated with TLR2 RNAi showed a drastic reduction in tumor volume.^[34]^ In some cases, TLR2 paired with TLR1 or TLR6 to form heterodimers to expand ligand spectrum. Lewis lung carcinoma (LLC) activated macrophage to produce IL-6 and TNF-а through activation of TLR2 and TLR6. TLR2/TLR6 complexes and the induced cytokines by macrophages further promoted LLC metastasis.^[23,32]^

The endotoxin level, especially lipopolysaccharide (LPS) in veins of hepatocellular carcinoma (HCC) patients, is much higher than normal. Jing et al found high expression of TLR4 in HCC tissues was strongly associated with poor prognosis in patients. TLR4 signaling induced by LPS could significantly increase tumor invasion and induce epithelia-mesenchymal transition in HCC cells.^[35]^ Dapito et al found translocation of intestinal microbiota and activation of TLR4 in liver cells promoted HCC through mediating increased proliferation and antiapoptosis.^[36]^ LPS promoted proliferation and prevented apoptosis in cevical cancer line Hela, via activating TLR4 and inducing the production of IL-6 and TGF-β1.^[37]^ In contrast, Hsiao and colleagues showed that endogenous TLR4 was overexpressed in hapatolastoma (HB) cells and TLR4 agonist inhibited tumor progression of HB cells in vitro. TLR4 signaling activated by LPS, dramatically decreased the transcripts of cytokine IL-8 and TNF-а, and downregulated the gelatinolytic activity of MMP-2, thus led to the decreased motility and invasiveness of HB cells.^[38]^

TLR7/8 ligand microRNA in blood of cancer patients can be detected as circulating biomarker. Tumor-secreted miR-21 and miR-29 act as paracrine agonist of TLRs to bind TLR7 in mouse or TLR8 in human immune cells, and ultimately lead to TLR-mediated tumor metastasis and growth. The secreted microRNA serves as a key regulator of tumor microenvironment and implicates its function in tumor-immune system communication, and thus represents a potential target for cancer treatment.^[39,40]^ Ochi agreed with these results, and believed that TLR7 signaling promoted carcinogenesis in mice and humans, and blockade of TLR7 protected host cells against tumor inflaming metastasis.^[41]^ These opposite outcomes in cancer development indicate the host specificity of TLR signaling controls the fate of cancer.

### 3.3 Targeting TLRs in cancer immunotherapy

**Figure 1:**
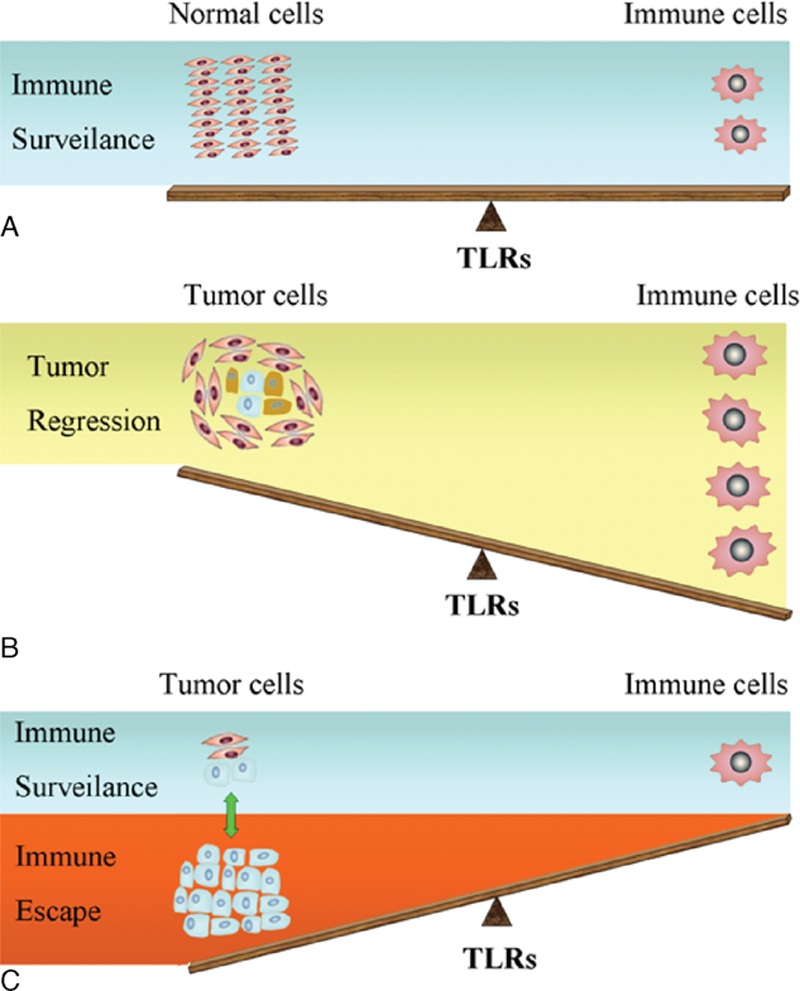
Relation of TLRs on immune cells and tumor cells to tumor immunotherapy is depicted. (A) TLRs of immune cells act as sensors in immune surveillance. (B) Sufficient immune cells recognize tumor antigens by TLRs and cause cell destruction through cell lysis, phagocytosis of dying cell, and cytokines secretion. (C) TLRs on tumor cells display different roles in the malignant process. Some TLRs on tumor cells facilitate immune escape, whereas other TLRs could terminate tolerant immune system and induce strong antitumor effects. TLRs = toll-like receptors.

TLRs of immune cells serve as sensors in immune surveillance. Immune cells recognize tumor antigens by TLRs, and infiltrate tumor stroma, which cause tumor destruction by direct lysis or cytokines secretion.^[42]^ However, TLRs on tumor cells may facilitate immune escape of tumor.^[43–45]^ Recent studies manifest different functions of TLRs on tumor cells. Activated TLRs in malignant process may play opposite roles: TLR signaling may promote cancer metastasis or kill tumor cells. Certain TLRs have been demonstrated to induce strong antitumor effects,^[46]^ and TLR signaling has been shown to enhance DC maturation and antigen presentation, which is one of the key issues in the effective tumor therapy. Some TLRs on tumor cells and immune cells have been considered as potential targets for antitumor immunotherapy to terminate tolerant immune system and kill tumor cells (Fig. 1). Thus, the potential of TLR agonists, as anticancer agents or vaccines, to induce effective immune reactions against tumor antigens has been exploited. Coley toxin (mixture of killed Streptococcus pyogenes and Serratia marcescens bacteria) and bacillus Calmette–Guerin (BCG) have become long-used anticancer drugs, which potently activate TLR2 and TLR4 signaling.^[47]^ TLR2 and TLR4 agonist, extract of larix leptolepis (ELL), activates bone marrow-derived dendritic cells (BMDCs) to induce the production of cytokines IL-12 and TNF-а, and induces tumor-specific cytotoxic T lymphocytes (CTLs) against cancer.^[48]^ TLR2 ligand HP-NAP (*Helicobacter pylori* neutrophil activating protein) is a potential therapeutic agent for nonmuscle invasive bladder cancer. HP-NAP is able to enhance the induction of the T helper 1 (TH1) cell differentiation and reduce vascularization of cancer through induction of IFN-γ.^[49]^ Lin et al found that TLR2 signaling in carcinogen diethylnitrosamine (DEN)-injured liver tissue induced intracellular senescence and activated autophagy to eliminate ROS accumulation and DNA damage, therefore, attenuated the development and progression of HCC. Accordingly, loss of TLR2 increased the susceptibility to DEN-induced hepatocellular carcinogenesis.^[50,51]^

Recent evidences suggested that TLR3 worked as a possible therapeutic target in many types of cancers. TLR3 signaling was activated in human pharyngeal cancer cell lines and oral sqaumous cell carcinoma cell lines and induced apoptosis of tumor cells by TLR3 ligand poly(I:C).^[52,53]^ Actually poly(I:C)-induced TLR3 signaling not only directly induced the apoptosis, but also destroyed tumor microenvironment by suppressing angiogenesis in human hepatocellular carcinoma cell lines MHCC97H and SMMC-7721.^[54]^ Moreover, Shime et al found activation of TLR3 by poly(I:C) converted tumor supporting macrophages to tumoricidal effectors in mice.^[55]^ Excitingly, novel strategies that target TLR3 to fight cancer have been emerging. Levitzki used chemical vectors attached to a specific ligand, such as antibody against EGFR in tumor cells, to introduce poly(I:C) into tumor cells. Upon the specific ligand binding receptor on the tumor cell surface, the poly(I:C)–ligand–receptor complex was internalized into cells. The internalized poly(I:C) activated TLR3, PKR, RIG-1, and MDA5 simultaneously. The simultaneous activation of these signaling proteins led to rapid death of tumor cells and bystander effects of secreted cytokines.^[56]^ Wang et al designed a novel immunotherapeutic method that based on cancer vaccine. In his study, poly(I:C)-DOTAP liposome complex nanoparticles were generated to enhance cellular penetration of poly(I:C) and consequential TLR3 signaling in BMDCs, by which the poly(I:C) nanoparticles augmented antitumor property of TLR3 signaling.^[57]^

The successful antitumor case of skin tumors that treated with imiquimod cream formulation showed that imiquimod acted as TLR7/8 agonist with antitumor properties. Drobits demonstrated that imiquimod treatment led to upregulation of chemokine CCL2 expression in TLR7/MyD88-dependent manner, which recruited plasmacytoid DCs (pDCs) and converted pDCs into tumor killer cells to eliminate tumor cells.^[58]^ Another TLR7 agonist 852A that stimulated pDCs to produce multiple cytokines has been conducting clinical study in patients with relapsed hematologic malignancies.^[59]^ Besides pDCs, activation of both CD8^+^ T cells and NK cells by TLR7 agonist is also responsible for antitumor response.^[60]^ In fact, the timing of TLR7/8 stimulation and the profile of induced cytokines are crucial factors for effective immunotherapy of cancer. Nonreasonable treatment of TLR7/8 agonist may lead to TLR7/8 tolerance in DC cells and inhibit the secretion of proinflammatory cytokines.^[61]^

Transcription activity and protein level of TLR9 were downregulated in pDCs and macrophages of patients with HBV-associated HCC. TLR9 signaling in HBV-HCC patients was also interfered by blocking MyD88-IRAK4 and IRF7 to produce IFN-a. Thus, researchers have realized that it is worth to focus strength on studying the escape mechanisms of HBV to interfere with TLR9 activity in HCC or chronic infection.^[62]^ In addition, the preclinical study has shown promise for a novel TLR-9 agonist C792 to treat multiple myeloma (MM), in which C792 significantly improves immune function and overcomes drug resistance in MM.^[63]^

Multiple TLR agonists have been considered for clinical application (Table 2). BCG has been approved for therapy of superficial bladder cancer, and conducting phase I studies in patients with colorectal cancer.^[64,65]^ TLR3 ligand IPH 31XX has been investigated in breast cancer patients.^[66]^ TLR4 agonist monophosphoryl lipid A (MPL) is in phase I clinical trial for patients with colorectal cancer, and approved for use in Cervarix vaccines as an adjuvant for the prophylaxis of HPV-associated cervical cancer.^[67]^ A phase II study of Flagellin-derived TLR5 agonist CBLB502 (Entolimod) in patients with advanced solid tumors is currently ongoing.^[68]^ TLR7 agonist imiquimod is approved for therapy of basal cell carcinoma.^[69]^ Phase II studies of another TLR7 agonist 852A is also investigated in melanoma patients.^[70]^ TLR9 agonist synthetic oligo-deoxynucleotide-expressing CpG motifs (CpG-ODN) can exert antitumor effects by blocking angiogenesis and enhance the antitumor activity of chemotherapy and radiation therapy in clinical studies.^[63]^ Unlike TLR agonists, TLR antagonists currently under development are anti-TLR antibodies and inactive molecule analogs of agonists. Although agonists have been centered in clinical development activity of TLRs, the discovery of TLR antagonists appear quite promising for proinflammatory and protumor TLRs. Recently, most antitumor TLR antagonists have still been studied in preclinical models.

**Table 2:**
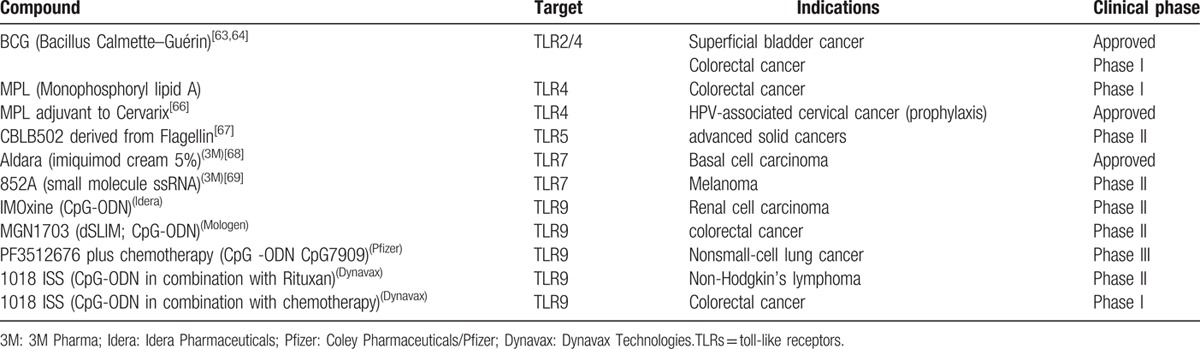
Clinical development: TLRs in cancer immunotherapy.

### 3.4 Synergistic application of TLRs and other cancer therapies

Radiation therapy is a conventional antitumor strategy which damages DNA of exposed tissue and provides multiple tumor antigens, but without systemic immunosuppression to cancer cells. However, TLR9 agonist activates B cells and pDCs in mice, and shows a potent immunostimulatory effect. Combination therapy with TLR9 agonist and radiation is a promising strategy of cancer treatment. Experiments showed that the CpG ODN plus radiotherapy augmented radiation efficacy reduced metastases of tumor cells and improved survival in mouse model of lung cancer.^[71,72]^ In addition to TLR9 agonist, engineered flagellin derivative TLR5 agonist CBLB502 was also found to protect mice and monkeys from acute radiation symptoms and improve radiation efficacy of tumor cells in radiotherapy.^[27,73]^ This implied that TLR5 agonist CBLB502 might be potential as efficient adjuvant for cancer radiotherapy.

Combined treatment of poly(I:C) or poly(A:U) and chemotherapeutics to tumor cells has much higher growth-inhibitory effect compared with single application. The combined application used in cancer therapy would decrease the clinical dosage of chemotherapeutics, which led to fewer side effects to patients.^[23,32,33,74]^

Oncolytic virus is an effective tool for cancer treatment. TLR9 activation could enhance antitumor immune response of oncolytic virus. The antitumor effect of oncolytic virus is significantly increased after insertion of TLR9 ligand repeated CpG island into the genome of virus. The advantage of oncolytic virus-CpG island is dependent on TLR9 and natural killer cells. The engineered oncolytic virus-CpG also increases the activation of antigen-specific T cells and decreases activation of myeloid-derived suppressor cells in mice.^[75]^

### 3.5 Potential mechanism of TLRs in cancer therapy

Recent advance in the field of TLRs research shows the therapeutic possibility on TLRs against cancer. However, the molecular mechanism of TLRs in cancer cells is still the biggest obstacle for clinical application. Besides the key role of TLRs in innate immunity, some groups have uncovered that the role of TLR signaling related with induction of autophagy, apoptosis or pyroptosis of cancer cells.^[29,76–78]^

**Figure 2:**
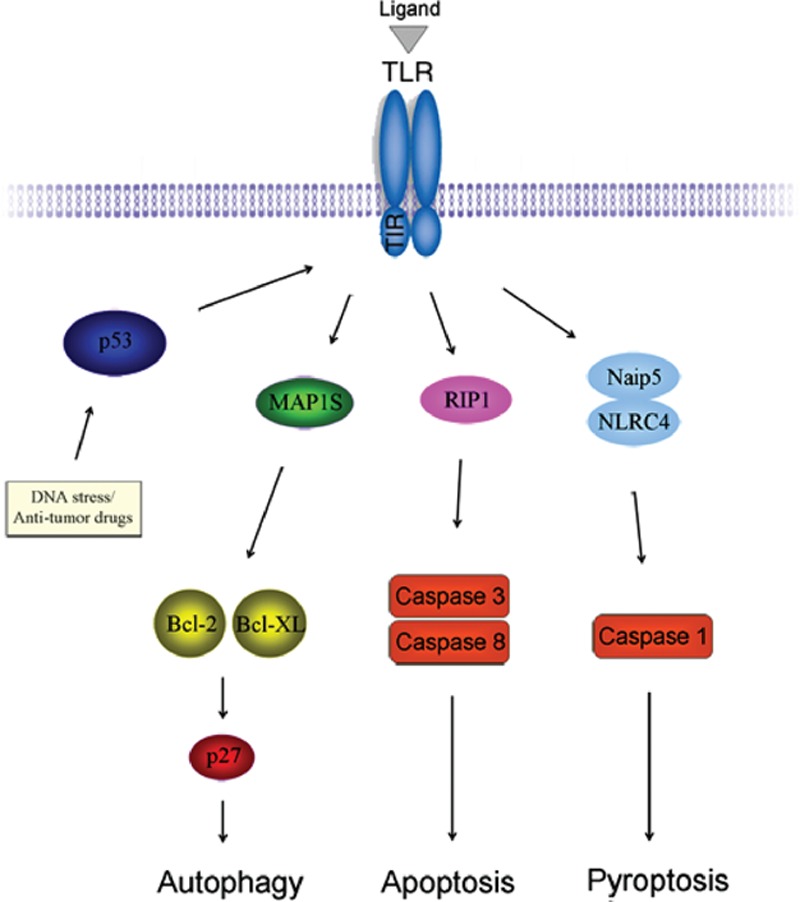
A model describing the mechanism of TLRs in cancer therapy. Activation of p53, due to DNA stress or antitumor agents, leads to enhance TLR signaling. Activated TLRs induce autophagy through recruitment of MAP1S and regulation of Bcl-2/XL and p27 in a noncanonical pathway. TLR3 signaling requires RIP1 to activate caspase-3 and caspase-8 and induces apoptosis. TLR5 ligand flagellin induces pyroptosis through introduction into cells by TLR5, and recognition by Naip5/NLRC4 to activate caspase-1. TLRs = toll-like receptors.

Apoptosis is the most efficient TLR-mediated programmed cell death (PCD), which involves activation of catabolic enzymes that lead to cell death through destruction of cell organelles.^[79]^ Estornes et al and Salaun et al illustrated the mechanism of TLR-induced apoptosis that TLR3 ligand dsRNA-induced cell death was through apoptosis directly, which required recruitment of RIP1, caspase-3 and caspase-8.^[52,80]^ Pyroptosis is a form of proinflammatory PCD and is initiated with the recognition of flagellin components. Several groups found that flagellin inhibited breast cancer through induction of caspase-1 activation-dependent pyroptosis, which was activated by the TLR5 and NLRC4/Naip5 signaling pathway.^[81–83]^ Autophagy is another classic TLR-mediated PCD, and it has dual and complicated roles in cancer. In response to nutrient starvation, autophagy produces recycled nutrients to avoid cell death, whereas high level of autophagy constitutes an alternative cell death pathway. Recent study of our lab and other groups showed that autophagy adaptor protein MAP1S involved in TLR signaling, and regulated Bcl-2/XL and p27 to activate autophagy through the noncanonical pathway.^[5,84–87]^ Current novel opinion on tumor suppressor p53 and TLRs showed that p53 modulated TLR signaling in cancer cells. The tumor suppressor p53, in response to stress signals or antitumor agents, induced transcriptional upregulation of individual TLR gene, therefore enhanced TLR downstream signaling in cancer cells (Fig. 2).^[53,88]^ Potential tumor suppressor MARVELD1 (MARVEL domain-containing 1), as a candidate regulator of TLR signaling, inhibits proliferation of cancer cells through regulating the expression of p53 and p16.^[89–93]^

## 4 Conclusion

Targeting toll-like receptors is now an exciting field for translational cancer research. Accumulating evidences indicate that expression of TLRs on tumor cells, which is known to mediate innate immune response, influences the proliferation and migration of tumor cells. Activation of different TLRs in cancer cells may play opposite role, antitumor or protumor. Therefore, better understanding the mechanism of TLRs in cancer biology will contribute to discovery of novel strategy for cancer therapy. Some small peptides and chemical compounds that were reported to work as agonists or antagonists for TLRs might be promising candidates for drugs against cancer. Toll-like receptor itself also has potential to be therapeutic target against cancer. Among these things, it opens door to the clinical application of TLRs for cancer therapy; however, it also puts the TLRs research to today's premier position.
